# Arrhythmogenic *KCNE* gene variants: current knowledge and future challenges

**DOI:** 10.3389/fgene.2014.00003

**Published:** 2014-01-24

**Authors:** Shawn M. Crump, Geoffrey W. Abbott

**Affiliations:** Bioelectricity Laboratory, Department of Pharmacology, Department of Physiology and Biophysics, School of Medicine, University of CaliforniaIrvine, CA, USA

**Keywords:** MinK-related peptide, MiRP, Long QT Syndrome, atrial fibrillation, Brugada Syndrome

## Abstract

There are twenty-five known inherited cardiac arrhythmia susceptibility genes, all of which encode either ion channel pore-forming subunits or proteins that regulate aspects of ion channel biology such as function, trafficking, and localization. The human *KCNE* gene family comprises five potassium channel regulatory subunits, sequence variants in each of which are associated with cardiac arrhythmias. *KCNE* gene products exhibit promiscuous partnering and in some cases ubiquitous expression, hampering efforts to unequivocally correlate each gene to specific native potassium currents. Likewise, deducing the molecular etiology of cardiac arrhythmias in individuals harboring rare *KCNE* gene variants, or more common *KCNE* polymorphisms, can be challenging. In this review we provide an update on putative arrhythmia-causing *KCNE* gene variants, and discuss current thinking and future challenges in the study of molecular mechanisms of *KCNE*-associated cardiac rhythm disturbances.

## Introduction

**Figure 1:**
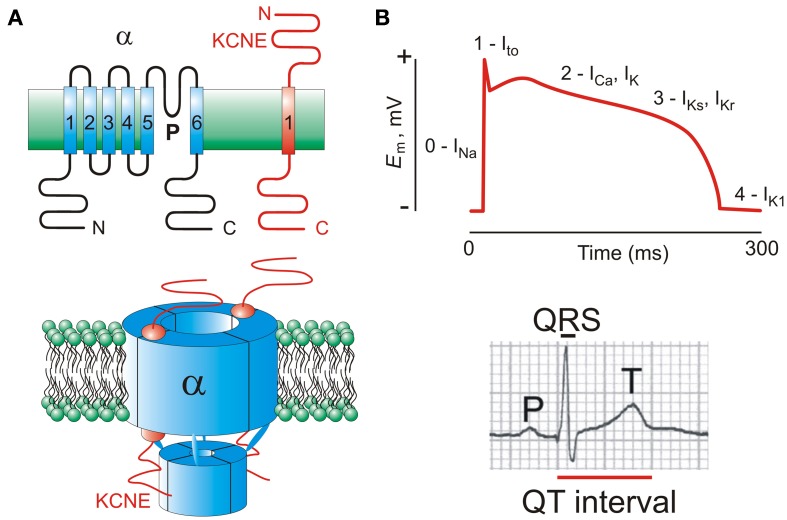
**KCNE subunits and the ventricular myocyte action potential. (A)** *Upper*, transmembrane topology of Kv α and KCNE subunits with transmembrane segments numbered; *lower*, one suggested stoichiometry of a KCNE-containing Kv channel complex. Extracellular side is uppermost in each case. **(B)** *Upper*, a ventricular action potential waveform indicating the major ionic currents that contribute to its morphology and duration; *lower*, a human surface ECG waveform showing the QT interval.

**Figure 2:**
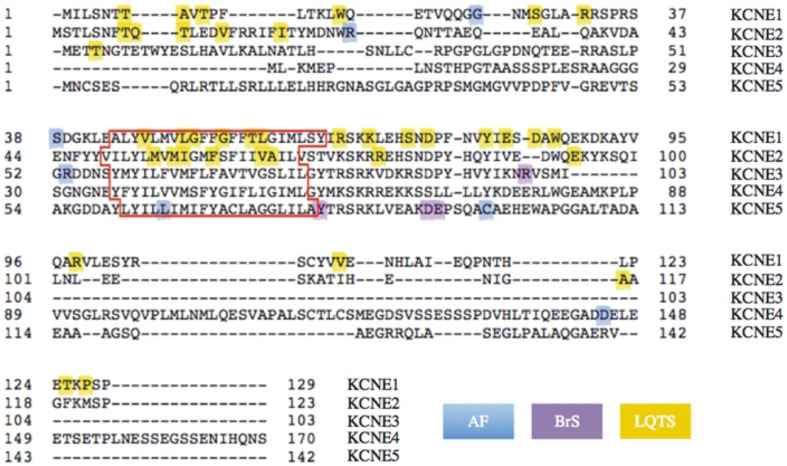
**Human KCNE1-KCNE5 protein sequence alignments and gene variants.** Image of aligned sequences generated using http://www.uniprot.org/align. Colors highlight inherited or sporadic non-synonymous mutations or polymorphisms resulting in single amino acid changes (changes involving >1 amino acid are omitted). In cases where an amino acid substitution is associated with LQTS in addition to another arrhythmia, only the latter is color-coded (see Table S1 for full information). The predicted transmembrane domain for each subunit is outlined in red.

A quarter of a century ago, Takumi and colleagues discovered a fraction of rat kidney mRNA that generated an unusual, slow-activating K^+^-selective current when injected into *Xenopus laevis* oocytes (Takumi et al., 1988). The protein product required for this slow current has been variously termed “minimal potassium channel” (MinK), “IsK” (for slow potassium current), and more recently KCNE1—the gene name *KCNE1* now being most commonly also used when referring to the protein product, for simplicity. We now know that KCNE1 is the founding member of a five-strong family of single transmembrane domain potassium channel ancillary (β) subunits (Figures 1, 2) that do not form currents alone but are essential for generation of some native K^+^ currents by virtue of formation of heteromeric ion channel complexes with voltage-gated potassium (Kv) channel pore-forming α subunits (Abbott and Goldstein, 1998). Because KCNE1 was relatively quickly found to be a molecular correlate of the slowly activating ventricular myocyte K^+^ current, I_Ks_ (Freeman and Kass, 1993), study of the KCNE family as a whole has historically been focused primarily on the heart. This is especially true for the study of the role of *KCNE* gene variants in human disease.

Although in the new millennium the role of various KCNE subunits in epithelia has been extensively explored, this work has been largely conducted using mouse models (Arrighi et al., 2001; Dedek and Waldegger, 2001; Barriere et al., 2003; Rivas and Francis, 2005; Roepke et al., 2006, 2009, 2011a,b; Preston et al., 2010). The existing evidence from human genetics of the necessity for KCNE proteins in extracardiac tissue (Schulze-Bahr et al., 1997; Tyson et al., 1997) probably represents the tip of the iceberg in terms of the actual importance of human KCNE proteins to tissues outside the heart, including polarized epithelia. In contrast, more than sixty *KCNE* gene variants have been suggested to associate with human cardiac arrhythmias. In this mini-review we summarize current knowledge on arrhythmia-associated KCNE gene variants and discuss the difficulties in establishing causality and molecular etiology when dealing with rare diseases and promiscuous regulatory proteins.

Kv channels play a central role in active repolarization of all excitable cells, including cardiac myocytes. In human ventricles, three types of Kv channel in particular are important for timely myocyte repolarization, and also for the action potential morphology optimal for rhythmic contractions, incorporating a plateau phase followed by relatively steep phase 3 repolarization (Figure 1B). During an action potential, membrane depolarization primarily from Na^+^ influx through voltage-gated Na^+^ channels is counteracted by a transient outward K^+^ (I_to_) current, producing the initial repolarization “notch.” Subsequently, slower delayed rectifier-generated outward K^+^ currents (I_Kr_ and I_Ks_) counteract inward Ca^2+^ flux through voltage-gated Ca^2+^ channels, modulating the strength of contraction and duration of the action potential plateau (Sanguinetti and Jurkiewicz, 1990; Niwa and Nerbonne, 2010).

## KCNE regulation of hERG: the α subunit underlying ventricular I_Kr_

The “rapidly activating” K^+^ current (I_Kr_) is the predominant human ventricular repolarization current under normal circumstances. I_Kr_ is generated by channels comprising a tetramer of hERG α subunits, encoded by the KCNH2 gene. *KCNH2* gene mutations are (together with *KCNQ1*) one of the top two identified inherited causes of the cardiac arrhythmia Long QT Syndrome (LQTS), which results from delayed ventricular myocyte repolarization, manifests as a prolonged electrocardiogram QT interval (Figure 1B), and can cause lethal ventricular fibrillation (Curran et al., 1995; Sanguinetti et al., 1995). hERG channels exhibit unusual properties that influence both cardiac electrical function and arrhythmogenesis. First, the majority of pathologic *KCNH2* gene mutations cause loss of function via protein maturation/trafficking defects rather than channel conduction or gating defects (Anderson et al., 2006). Second, upon membrane depolarization, hERG channels open and then rapidly inactivate. As the membrane begins to repolarize, hERG recovers rapidly from inactivation but deactivates slowly. This creates an atypical mode of inward rectification (classic inward rectifier K^+^ channels being generated instead by tetramers of two-transmembrane domain α subunits) (Smith et al., 1996). It ensures that hERG channels pass robust currents relatively late in the ventricular action potential, to speed phase 3 repolarization, without curtailing the preceding plateau phase. Third, hERG is highly susceptible to block by drugs from a wide range of chemical structures, making it the bane of pharmaceutical companies attempting to bring to market otherwise efficacious drugs that fail safety standards because they inhibit hERG and therefore are predicted (or demonstrated) to cause drug-induced LQTS (diLQTS) (Sanguinetti et al., 1995; Chen et al., 2002).

hERG channels are modulated by both KCNE1 and KCNE2 (originally named MinK-related Protein 1 or MiRP1) *in vitro* and potentially also in human heart (McDonald et al., 1997; Abbott et al., 1999). Currents generated by expression of hERG alone in heterologous expression systems recapitulate most of the functional properties of native I_Kr_, including the unusual (for an S4 family α subunit) inward rectification and the exquisite drug sensitivity (Sanguinetti et al., 1995). However, KCNE1 forms heteromeric complexes with hERG and increases hERG currents by an as yet unknown mechanism when the two proteins are co-expressed in COS cells (McDonald et al., 1997). In addition, inherited *KCNE1* mutants associated with human LQTS impair hERG function and/or trafficking (Table S1). These findings suggest that KCNE1 modulates hERG *in vivo* in at least some areas of the ventricle (Bianchi et al., 1999).

hERG also forms heteromeric complexes with KCNE2, when the two are co-expressed in Chinese Hamster Ovary (CHO) cells. When co-expressed in either *Xenopus* oocytes or CHO cells, KCNE2 alters hERG function, shifting the voltage dependence of activation, decreasing unitary conductance, and speeding deactivation. Importantly, inherited gene variants in human *KCNE2* that are associated with LQTS impair hERG gating, which would be predicted to delay ventricular repolarization as is seen in LQTS (Abbott et al., 1999) (Table S1). More strikingly, *KCNE2* gene variants associated with drug-induced LQTS (diLQTS) in some cases increase the sensitivity of hERG *in vitro* to block by the specific drug that precipitated the arrhythmic episode *in vivo* (Abbott et al., 1999; Sesti et al., 2000).

This is highly supportive of a role for KCNE2 in direct regulation of hERG channels in human ventricular myocardium. Indeed, KCNE2-rERG complexes have been isolated from rat heart and a plethora of other evidence suggests that KCNE2 regulates ERG in the hearts of several species (Jiang et al., 2004; McCrossan et al., 2009; Zhang et al., 2011). However, the debate that has surrounded the existence and necessity of hERG-KCNE2 complexes in human heart highlights the difficulties in nailing down the molecular correlates of multi-subunit channels. This problem is exacerbated when considering human heart, which compared to animal studies, involves more restrictions in tissue availability and less practicable experimental options. Temporal dynamism and spatial diversity in the makeup of these complexes (as almost certainly occurs with KCNE-containing channels) also stymies this research, as does the fact (as in the case of KCNE2-hERG) that the effects of KCNE2 on hERG are relatively subtle and may be expression system-dependent. Furthermore, when disease associations are relatively rare and the subunits involved exhibit promiscuous partnering, even human genetics does not automatically uncover the precise functional role of a regulatory subunit. Mouse models have been useful in discovering physiological and arrhythmogenic roles for KCNE subunits and their disruption (Temple et al., 2005; Roepke et al., 2008; Hu et al., 2013) but come with the caveat, especially for the heart, that there is a big divide between mouse and human heart in terms of physiology, the primary repolarizing currents, and their molecular underpinnings (Nerbonne et al., 2001).

## KCNE modulation of KCNQ1: the α subunit underlying human ventricular I_Ks_

KCNQ1 is the pore-forming subunit of cardiac I_Ks_, a slow-activating K^+^ current that, in human ventricles, may act primarily as a back-up for I_Kr_ when the latter is diminished by e.g., drug block, mutation, or during periods of increased heart-rate (Barhanin et al., 1996; Sanguinetti et al., 1996; Silva and Rudy, 2005). KCNQ1 is the endogenous *Xenopus laevis* oocyte α subunit that was awakened by injected KCNE1 in Takumi's original discovery of the KCNE family. It took a further 8 years before the cloning of human and *Xenopus* KCNQ1 (then termed KvLQT1) was reported (Barhanin et al., 1996; Sanguinetti et al., 1996), KCNQ1 was linked to LQTS (Wang et al., 1996), and the KCNE1 functionality and I_Ks_ molecular correlate conundrums solved.

Or were they? We know for certain that ventricular KCNQ1-KCNE1 complexes exist in the hearts of some large mammals, and almost certainly contribute to human ventricular myocyte repolarization. Loss-of-function gene variants in either gene reduce repolarizing force and delay ventricular repolarization, causing LQTS, particularly manifest (for KCNQ1 mutants) during periods of sympathetic stimulation such as while swimming (Wang et al., 1996; Tyson et al., 1997; Ackerman et al., 1999).

However, it is also highly likely that other KCNQ1-KCNE channels help to repolarize some ventricular myocytes in human heart, and that KCNE1 regulates other Kv α subunits in human heart as well. This means that KCNE1-associated LQTS (termed LQT5) could be much more complicated than just disruption of ventricular KCNQ1-KCNE1. Another fly in the ointment for those wishing to rationalize the genetics of ventricular arrhythmias is that KCNQ1-hERG complexes almost certainly exist in human heart and loss-of-function variants in either subunit may affect the function of the other (Ehrlich et al., 2004; Ehrlich, 2010; Ren et al., 2010). Add to this the notion that different KCNEs may participate in the same multi-subunit channel complex with KCNQ1 (Wu et al., 2006) and the riddle that is the molecular etiology of KCNE-associated arrhythmogenesis becomes ever more enigmatic. KCNQ1 can be modulated by all five known human KCNEs, with diverse functional outcomes (McCrossan and Abbott, 2004). The stoichiometry of KCNQ1-KCNE complexes is still under debate, but there are almost certainly four KCNQ1 α subunits in complex with 2–4 KCNE1 subunits (and possibly a variable number of KCNE1 subunits within these limits, depending upon expression levels) (Chen et al., 2003; Nakajo et al., 2010; Yu et al., 2013).

KCNE1 slows KCNQ1 activation 5–10-fold, eliminates its inactivation, increases unitary conductance and tweaks ion selectivity and pharmacology (Sesti and Goldstein, 1998). Strikingly, KCNE1 also strongly modulates KCNQ1 affinity for PIP_2_ (an important regulatory factor) (Loussouarn et al., 2003), and KCNE1 mediates protein kinase C-stimulated clathrin-mediated endocytosis of KCNQ1-KCNE1 (Kanda et al., 2011). KCNE1 also regulates KCNQ1 in the inner ear, which is why some individuals harboring loss-of-function mutants in KCNQ1 or KCNE1 in both alleles succumb to Jervell and Lange-Nielsen Syndrome (JLNS), comprising both LQTS and sensorineural deafness. Rather than mediating repolarization, in the inner ear KCNQ1-KCNE1 probably serves primarily to maintain a K^+^-rich environment in the endolymph (Wangemann et al., 1995; Vetter et al., 1996; Wangemann et al., 1996).

KCNE2 performs electrical alchemy with KCNQ1, converting it into a constitutively active channel that has lost much of its voltage dependence (Tinel et al., 2000). KCNQ1-KCNE2 is especially important in various polarized epithelia (Abbott, 2012) but could also be present in human heart. Some atrial fibrillation (AF)-associated KCNQ1 and KCNE2 human gene variants augment KCNQ1-KCNE2 currents (which are typically much smaller in terms of outward current even than KCNQ1 alone) and would therefore be predicted to shorten the atrial action potential, thought to predispose to AF (Yang et al., 2004) (Table S1). KCNE3, one variant of which also associates with AF (Zhang et al., 2005) has broadly similar effects on KCNQ1, but the resultant currents are much larger than those of KCNQ1-KCNE2 and important in different epithelial cells to those of KCNQ1-KCNE2 (Schroeder et al., 2000). Either heteromer type could contribute to background K^+^ currents in human cardiomyocytes but this has yet to be established. Similarly, KCNE2 and KCNE3 could each contribute to tripartite complexes with KCNQ1 and KCNE1, or to variegated macromolecular assemblies also including hERG, with potentially complex and dynamic functional attributes. Understanding the expression and regulation of such complexes *in vivo* is challenging but could lead to development of anti-arrhythmic drugs with, for example, improved spatial selectivity (Yu et al., 2013).

KCNE4 inhibits KCNQ1 and may potentially serve this function *in vivo* in human heart (Grunnet et al., 2002, 2005). KCNQ1-KCNE5 complexes generate currents superficially similar to those of KCNQ1-KCNE1, but with much more positive activation voltages (Angelo et al., 2002), suggesting either an inhibitory role for KCNE5 (originally termed KCNE1L), or perhaps an as-yet not understood role in more populous complexes with other KCNEs. KCNE4 and KCNE5 gene variants that dampened their inhibitory effects on KCNQ1 (gain-of-function mutants) also associate with AF (Ravn et al., 2008; Ohno et al., 2011) (Table S1).

## KCNE modulation of the Kv4 α subunits underlying human ventricular I_to_

Human ventricular cardiomyocyte action potentials exhibit a sharp peak and a notch, as depolarization primarily by Na^+^ influx is curtailed abruptly by K^+^ exiting through rapidly activating Kv channels. The channels whose primary task is to stem phase 0 depolarization are, in human heart, the Kv4.2 and Kv4.3 channels, via generation of the transient outward Kv current (I_to_). This current is transient because both channel types also inactivate rapidly. KCNE subunits regulate Kv4.2 and Kv4.3 when co-expressed *in vitro*, and it is thought that this type of regulation also occurs in human heart (Zhang et al., 2001; Niwa and Nerbonne, 2010).

Kv4 channels are also regulated by the cytoplasmic KChIP2 subunit *in vivo*, and KCNEs can regulate Kv4-KChIP2 complexes. KCNE1 and KCNE3-5 subunits each accelerate Kv4.3-KChIP2 inactivation, while KCNE2 slows inactivation and induces an overshoot of inactivation recovery *in vitro* in CHO cells, similar to that observed in human heart for I_to_. KCNE2 augments Kv4.2 current by mechanisms including slowing of inactivation (Radicke et al., 2006); although also slowing Kv4.3 inactivation, KCNE2 reduces its peak current *in vitro* (Liu et al., 2006; Wu et al., 2010). KCNE subunits also modulate Kv4 channel pharmacology and temperature sensitivity (Radicke et al., 2008, 2009, 2013).

Inherited loss-of-function sequence variants in the cardiac Nav1.5 channel α subunit gene (*SCN5A*) are the most common identified genetic cause of Brugada Syndrome (BrS), a lethal ventricular arrhythmia (Brugada et al., 2006). More recently, BrS-associated *KCNE* gene variants have emerged that augment Kv4 currents, mimicking *SCN5A* loss of function (Delpon et al., 2008; Ohno et al., 2011; Nakajima et al., 2012) (Table S1). These are rare, and like the majority of *KCNE* arrhythmia-associated gene variants, are not backed by familial analyses or the statistical confidence that comes with prevalence in large cohorts. However, they have been given some credence in the field, largely because their effects *in vitro* are consistent with what would be predicted from work with *SCN5A* (which is strongly statistically linked to BrS), and they occur in BrS patients lacking *SCN5A* gene mutations.

## Arrhythmogenic *KCNE* gene variants: two decades of discovery

The first *KCNE* gene variant identified in the human population was the *KCNE1* S38G polymorphism (Lai et al., 1994). The majority of people harbor one S and one G allele, with the 38GG genotype being next most common, and the 38SS being the least common (~10%, depending on the population studied). S38G genotype reportedly influences predisposition to both AF and LQTS (Fatini et al., 2006; Prystupa et al., 2006; Xu et al., 2008; Husser et al., 2009), and heart failure (Fatini et al., 2010), depending on factors including sex, age, BMI, diabetes, fibrinogen, hypercholesterolemia, hypertension, and another *KCNE1* SNP (Friedlander et al., 2005).

The first *KCNE* gene variant to be identified as pathologic was D76N, a dominant-negative mutation that causes JLNS (Schulze-Bahr et al., 1997) and impairs KCNQ1-KCNE1 current by a combination of reduced unitary conductance and impaired gating. This was closely followed by discovery of S74L, which like D76N shifts the voltage dependence of activation and accelerates KCNQ1-KCNE1 channel closing (Splawski et al., 1997; Sesti and Goldstein, 1998).

The next *KCNE* gene to be associated with cardiac arrhythmia was *KCNE2*, with discovery of the rare M54T, I57T, A116V loss-of-function mutants, and the T8A and Q9E polymorphisms (Abbott et al., 1999; Sesti et al., 2000). T8A, harbored by >1% of US Caucasians, is pathogenic only in combination with drug interaction, as it has no apparent effects without drug but results in loss of a glycosylation site that shields hERG-KCNE2 channels from block by sulfamethoxazole (Sesti et al., 2000; Park et al., 2003). Q9E, represented in 1–2% of African Americans, impairs channel function slightly without drug, and also increases sensitivity of hERG-KCNE2 to block by macrolide antibiotics (Abbott et al., 1999; Ackerman et al., 2003).

Following these findings and the cloning of *KCNE3-5*, gene variants in all five *KCNE* genes have been associated with LQTS, BrS, and/or AF (Table S1). *KCNE3* variants are implicated in AF, BrS6, and LQTS (Zhang et al., 2005; Delpon et al., 2008; Ohno et al., 2009). *KCNE4* E45D augments KCNQ1-KCNE4 current and was discovered in a Chinese patient with AF (Zeng et al., 2007). *KCNE5* gene variants include AF-associated L65F and BrS/idiopathic ventricular fibrillation-associated Y81H and D92E/E93X (Ohno et al., 2011). In contrast, the *KCNE5*-C97T polymorphism may be protective against AF (Ravn et al., 2005).

Reported *KCNE* sequence variants in arrhythmias offer as yet scant information on which to make concrete links between types/positions of sequence variants and classes of arrhythmia. However, some patterns emerge when contemplating the 49 variants that fall into the category of point mutants causing single amino acid changes (Figure 2), of the 59 reported *KCNE* variants for which we consider sufficient evidence is available that they at least be seriously considered as having a pathogenic role in the heart (Table S1). These are variants reported absent in control patients sequenced in the same study, and/or those for which cellular electrophysiology is consistent with disease association. For KCNE1 and KCNE2, pathogenic mutations in the transmembrane domain occur with a periodicity suggestive of the known α-helicity of this region, perhaps indicating disruption of the face most important to α subunit gating. BrS-associated KCNE variants cluster on the intracellular side; AF-associated variants in KCNE1-3 cluster in the extracellular region, whereas the opposite is true for KCNE4 and 5 (Figure 2).

To conclude, KCNE proteins are essential for normal cardiac function, and human genetics studies are essential in our understanding of this. However, human *KCNE* gene variants are mostly quite rare and typically lacking familial linkage, therefore some of the listed variants come into the category of genetic noise—variants that happen to be found in patients but do not contribute to the arrhythmia (Kapplinger et al., 2013). Also, in addition to their partnering promiscuity, all *KCNE* genes are expressed in multiple extracardiac tissues (McCrossan and Abbott, 2004). Therefore, *KCNE* sequence variants may manifest in multiple tissues, and these pathologies could indirectly impact cardiac function both electrically and structurally, further complicating our efforts to comprehend *KCNE*-related cardiac diseases. Comparatively little is known of the extracardiac effects of human *KCNE* gene disruption: the KCNE1-linked inner ear defect in JLNS, and genome-wide association studies showing statistical linkage to early-onset myocardial infarction and reduced lung capacity (Kathiresan et al., 2009; Soler Artigas et al., 2011). In contrast, mouse *Kcne* genes are known to be important in, e.g., the kidneys, adrenals, stomach, colon, airways, and thyroid (Arrighi et al., 2001; Dedek and Waldegger, 2001; Barriere et al., 2003; Rivas and Francis, 2005; Roepke et al., 2006, 2009, 2011a,b; Preston et al., 2010), and in some cases dysfunction of these tissues has been demonstrated to negatively impact the heart (Roepke et al., 2009; Hu et al., 2013, 2014). *Kcne* knockout mouse studies are providing invaluable inroads into the maze of *KCNE* physiology and disease, and constitute a substrate for future human genetics studies necessary for extrapolation of mouse data to human systems.

## Conflict of interest statement

The authors declare that the research was conducted in the absence of any commercial or financial relationships that could be construed as a potential conflict of interest.

## Abbreviations

AF: atrial fibrillation
BrS: Brugada syndrome
CHO: Chinese hamster ovary
diLQTS: drug-induced Long QT Syndrome
I_*Ks*_: cardiac-delayed rectifier-like K^+^ current
JLNS: Jervell and Lange-Nielsen syndrome.
