# Supplementary material for: Arrhythmogenic KCNE gene variants: current knowledge and future challenges
Source: Front Genet. 2014 Jan 24;5:3. doi: 10.3389/fgene.2014.00003 (PMC3900874; doi:10.3389/fgene.2014.00003)
Supplement: Supplementary file 1 [file DataSheet1.DOCX]

| *Gene* | Mutation | Disease | *In vitro* cellular effects | Reference |
| --- | --- | --- | --- | --- |
| *KCNE1* | T7I | JLNS | n.r. | ([Schulze-Bahr, Wang et al. 1997](#_ENREF_33)) |
| *KCNE1* | A8V | LQT5, AF | I_Ks_ +9 mV positive shift in V_1/2_ activation. No change in deactivation or activation time constants. I_Kr_ loss of function. | ([Ohno, Zankov et al. 2007](#_ENREF_25), [Kapplinger, Tester et al. 2009](#_ENREF_14)) |
| *KCNE1* | T10M | LQT5 | n.r. | ([Kapplinger, Tester et al. 2009](#_ENREF_14)) |
| *KCNE1* | W17X | LQT5 | n.r. | ([Kapplinger, Tester et al. 2009](#_ENREF_14)) |
| *KCNE1* | G25V | Lone AF | I_Ks_ gain of function | ([Olesen, Bentzen et al. 2012](#_ENREF_26)) |
| *KCNE1* | S28L | LQT5, AV block (neonatal) | n.r. | ([Shim, Ito et al. 2005](#_ENREF_36), [Kapplinger, Tester et al. 2009](#_ENREF_14)) |
| *KCNE1* | R32H | LQT5 | Gating not altered | ([Splawski, Shen et al. 2000](#_ENREF_37), [Westenskow, Splawski et al. 2004](#_ENREF_39), [Kapplinger, Tester et al. 2009](#_ENREF_14)) |
| *KCNE1* | 38G versus 38S | AF, LQT5 | I_Ks_ loss of function, reduced membrane K_V_7.1 (KvLQT1) channel | ([Ehrlich, Zicha et al. 2005](#_ENREF_6)) ([Fatini, Sticchi et al. 2006](#_ENREF_7), [Prystupa, Dzida et al. 2006](#_ENREF_29), [Xu, Yang et al. 2008](#_ENREF_41), [Husser, Stridh et al. 2009](#_ENREF_11)) |
| *KCNE1* | V47F | LQT5 | HERG Gain of Function | ([Bianchi, Shen et al. 1999](#_ENREF_3)) |
| *KCNE1* | L51H | LQT5 | Does not process properly, not found in membrane | ([Bianchi, Shen et al. 1999](#_ENREF_3)) |
| *KCNE1* | G52R | LQT5 | I_Ks_ reduced 50% | ([Ma, Lin et al. 2003](#_ENREF_17)) |
| *KCNE1* | G55S | LQT5 | n.r. | ([Kapplinger, Tester et al. 2009](#_ENREF_14)) |
| *KCNE1* | T58P | LQT5 | n.r. | ([Kapplinger, Tester et al. 2009](#_ENREF_14)) |
| *KCNE1* | L59P | LQT5 | n.r. | ([Kapplinger, Tester et al. 2009](#_ENREF_14)) |
| *KCNE1* | G60D | Lone AF | I_Ks_ gain of function; faster deactivation | ([Olesen, Bentzen et al. 2012](#_ENREF_26)) |
| *KCNE1* | R67C | LQT5 | n.r. | ([Kapplinger, Tester et al. 2009](#_ENREF_14)) |
| *KCNE1* | R67H | LQT5 | n.r. | ([Kapplinger, Tester et al. 2009](#_ENREF_14)) |
| *KCNE1* | K70M | LQT5 | n.r. | ([Kapplinger, Tester et al. 2009](#_ENREF_14)) |
| *KCNE1* | K70N | LQT5 | n.r. | ([Lai, Su et al. 2005](#_ENREF_15)) |
| *KCNE1* | S74L  D76N | LQT5 (JLNS+RW) | D76N: I_Ks_ - smaller unitary currents and open probabilities. Dominant negative.  I_Kr_ – loss of function.  S74L: I_Ks_: loss of function. | ([Splawski, Tristani-Firouzi et al. 1997](#_ENREF_38))  ([Duggal, Vesely et al. 1998](#_ENREF_5))  ([Sesti and Goldstein 1998](#_ENREF_35))  ([Bianchi, Shen et al. 1999](#_ENREF_3)) |
| *KCNE1* | N75-fs+34X | LQT5 | n.r. | ([Kapplinger, Tester et al. 2009](#_ENREF_14)) |
| *KCNE1* | Y81C | LQT5? | I_Ks_ loss of function, Positive shift for voltage of activation | ([Wu, Lai et al. 2006](#_ENREF_40)) |
| *KCNE1* | E83K | LQT5 | n.r. | ([Kapplinger, Tester et al. 2009](#_ENREF_14)) |
| *KCNE1* | D85N | LQT5?  DiLQTS  DiTdP | No effect on I_Ks_, V_1/2_ of activation, or deactivation properties | ([Paulussen, Gilissen et al. 2004](#_ENREF_27), [Gouas, Nicaud et al. 2005](#_ENREF_9), [Zeng, Pu et al. 2005](#_ENREF_44), [Nielsen, Winkel et al. 2007](#_ENREF_21), [Zeng, Tan et al. 2007](#_ENREF_43), [Nishio, Makiyama et al. 2009](#_ENREF_22), [Porthan, Marjamaa et al. 2010](#_ENREF_28), [Kaab, Crawford et al. 2012](#_ENREF_13), [Lin, Horigome et al. 2012](#_ENREF_16)) |
| *KCNE1* | W87R | LQT5 | I_Ks_ loss of function, altered gating | ([Bianchi, Shen et al. 1999](#_ENREF_3)) |
| *KCNE1* | R98W | LQT5? | Disrupts I_Ks_ trafficking, right-shifts voltage dependence of activation | ([Harmer, Wilson et al. 2010](#_ENREF_10)) |
| *KCNE1* | V109I | LQT5 | I_Ks_ loss of function (36%) | ([Schulze-Bahr, Schwarz et al. 2001](#_ENREF_32)) |
| *KCNE1* | Q117X | LQT5 | n.r. | ([Kapplinger, Tester et al. 2009](#_ENREF_14)) |
| *KCNE1* | T125M | LQT5 | n.r. | ([Kapplinger, Tester et al. 2009](#_ENREF_14)) |
| *KCNE1* | P127T | LQT5, RWS | n.r. | ([Splawski, Shen et al. 2000](#_ENREF_37)) |
| *KCNE2* | T8A  Q9E | LQT6, diLQTS | Increases drug sensitivity of I_Kr_ | ([Abbott, Sesti et al. 1999](#_ENREF_1)) ([Sesti, Abbott et al. 2000](#_ENREF_34)) |
| *KCNE2* | T10M | LQT6 | I_Kr_ slow inactivation recovery and inactivation | ([Gordon, Panaghie et al. 2008](#_ENREF_8)) |
| *KCNE2* | V14I | LQT6 | n.r. | ([Kapplinger, Tester et al. 2009](#_ENREF_14)) |
| *KCNE2* | I20N | LQT6 | n.r. | ([Kapplinger, Tester et al. 2009](#_ENREF_14)) |
| *KCNE2* | R27C | AF | I_Q1-E2_ Gain-of-function | ([Yang, Xia et al. 2004](#_ENREF_42)) |
| *KCNE2* | R27H | LQT6 | n.r. | ([Kapplinger, Tester et al. 2009](#_ENREF_14)) |
| *KCNE2* | 52-54 deletion | LQT6 | n.r. | ([Napolitano, Priori et al. 2005](#_ENREF_20)) |
| *KCNE2* | M54T  I57T  A116V | LQT6, diLQTS | Pathological I_Kr_ loss of function ± drug. M54T and I57T also slow K_V_2.1 activation. | ([Abbott, Sesti et al. 1999](#_ENREF_1), [Sesti, Abbott et al. 2000](#_ENREF_34), [Kapplinger, Tester et al. 2009](#_ENREF_14)) |
| *KCNE2* | M56V | LQT6 | n.r. | ([Napolitano, Priori et al. 2005](#_ENREF_20)) |
| *KCNE2* | F60L | LQT6 | n.r. | ([Millat, Chevalier et al. 2006](#_ENREF_18)) |
| *KCNE2* | V65 M | LQT6, Syncope | I_Kr_ loss of function, accelerated inactivation time course | ([Isbrandt, Friederich et al. 2002](#_ENREF_12)) |
| *KCNE2* | V65L | LQT6 | n.r. | ([Kapplinger, Tester et al. 2009](#_ENREF_14)) |
| *KCNE2* | A66V | LQT6? | n.r. | ([Ackerman, Tester et al. 2003](#_ENREF_2)) |
| *KCNE2* | R77W | LQT6? | n.r. | ([Millat, Chevalier et al. 2006](#_ENREF_18)) |
| *KCNE2* | R77Q | LQT6 | n.r. | ([Kapplinger, Tester et al. 2009](#_ENREF_14)) |
| *KCNE2* | E94G | LQT6 | n.r. | ([Kapplinger, Tester et al. 2009](#_ENREF_14)) |
| *KCNE2* | P123-fs+14x | LQT6 | n.r. | ([Kapplinger, Tester et al. 2009](#_ENREF_14)) |
| *KCNE3* | T4A | LQTS?  BrS | No effect on I_Q1-E3_  I_to_ gain of function | ([Ohno, Toyoda et al. 2009](#_ENREF_23))  ([Nakajima, Wu et al. 2012](#_ENREF_19)) |
| *KCNE3* | R53H | AF | I_Q1-E3_ gain of function | ([Zhang, Liang et al. 2005](#_ENREF_45)) |
| *KCNE3* | R99H | BrS  LQTS? | K_V_4.3, I_to_ gain of function.  I_Q1-E3_ Loss of function | ([Delpón, Cordeiro et al. 2008](#_ENREF_4))  ([Ohno, Toyoda et al. 2009](#_ENREF_23)) |
| *KCNE4* | E145D | AF | I_Q1-E4_ gain of function | ([Zeng, Tan et al. 2007](#_ENREF_43)) |
| *KCNE5* | L65F | AF | I_Ks_ gain of function | ([Ravn, Aizawa et al. 2008](#_ENREF_30)) |
| *KCNE5* | Y81H  D92E;E93X | BrS/idiopathic VF | K_V_4.3 gain of function;  no change in I_Q1-E5_ density | ([Ohno, Zankov et al. 2011](#_ENREF_24)) |
| *KCNE5* | C97T | AF | n.r. | ([Ravn, Hofman-Bang et al. 2005](#_ENREF_31)) |

**Table S1. *KCNE* gene variants associated with cardiac arrhythmia.**

AF, Atrial Fibrillation; AV block, atrioventricular block; BrS, Brugada Syndrome; diLQTS, drug induced Long QT Syndrome; diTdP, drug induced torsade de pointes; fs, frame shift; JLNS, Jervell and Lange-Nielsen Syndrome; n.r., not reported; RWS, Romano-Ward Syndrome; VF, ventricular fibrillation. Sources include cited papers, <http://www.genomed.org/lovd2/home.php> and <http://www.fsm.it/cardmoc/>. Gene accession numbers: KCNE1, P15382; KCNE2, Q9Y6J6; KCNE3, Q9Y6H6; KCNE4, Q8WWG9; KCNE5, Q9UJ90.

**ReferenceS**

Abbott, G. W., F. Sesti, I. Splawski, M. E. Buck, M. H. Lehmann, K. W. Timothy, M. T. Keating and S. A. Goldstein (1999). "MiRP1 forms IKr potassium channels with HERG and is associated with cardiac arrhythmia." Cell **97**(2): 175-187.

Ackerman, M. J., D. J. Tester, G. S. Jones, M. L. Will, C. R. Burrow and M. E. Curran (2003). "Ethnic differences in cardiac potassium channel variants: implications for genetic susceptibility to sudden cardiac death and genetic testing for congenital long QT syndrome." Mayo Clin Proc **78**(12): 1479-1487.

Bianchi, L., Z. Shen, A. T. Dennis, S. G. Priori, C. Napolitano, E. Ronchetti, R. Bryskin, P. J. Schwartz and A. M. Brown (1999). "Cellular Dysfunction of LQT5-MinK Mutants: Abnormalities of IKs, IKr and Trafficking in Long QT Syndrome." Human Molecular Genetics **8**(8): 1499-1507.

Delpón, E., J. M. Cordeiro, L. Núñez, P. E. B. Thomsen, A. Guerchicoff, G. D. Pollevick, Y. Wu, J. K. Kanters, C. T. Larsen, E. Burashnikov, M. Christiansen and C. Antzelevitch (2008). "Functional Effects of KCNE3 Mutation and Its Role in the Development of Brugada Syndrome / CLINICAL PERSPECTIVE." Circulation: Arrhythmia and Electrophysiology **1**(3): 209-218.

Duggal, P., M. R. Vesely, D. Wattanasirichaigoon, J. Villafane, V. Kaushik and A. H. Beggs (1998). "Mutation of the Gene for IsK Associated With Both Jervell and Lange-Nielsen and Romano-Ward Forms of Long-QT Syndrome." Circulation **97**(2): 142-146.

Ehrlich, J. R., S. Zicha, P. Coutu, T. E. Hebert and S. Nattel (2005). "Atrial fibrillation-associated minK38G/S polymorphism modulates delayed rectifier current and membrane localization." Cardiovasc Res **67**(3): 520-528.

Fatini, C., E. Sticchi, M. Genuardi, F. Sofi, F. Gensini, A. M. Gori, M. Lenti, A. Michelucci, R. Abbate and G. F. Gensini (2006). "Analysis of minK and eNOS genes as candidate loci for predisposition to non-valvular atrial fibrillation." Eur Heart J **27**(14): 1712-1718.

Gordon, E., G. Panaghie, L. Deng, K. J. Bee, T. K. Roepke, T. Krogh-Madsen, D. J. Christini, H. Ostrer, C. T. Basson, W. Chung and G. W. Abbott (2008). "A KCNE2 mutation in a patient with cardiac arrhythmia induced by auditory stimuli and serum electrolyte imbalance." Cardiovasc Res **77**(1): 98-106.

Gouas, L., V. Nicaud, M. Berthet, A. Forhan, L. Tiret, B. Balkau and P. Guicheney (2005). "Association of KCNQ1, KCNE1, KCNH2 and SCN5A polymorphisms with QTc interval length in a healthy population." Eur J Hum Genet **13**(11): 1213-1222.

Harmer, S. C., A. J. Wilson, R. Aldridge and A. Tinker (2010). "Mechanisms of disease pathogenesis in long QT syndrome type 5." Am J Physiol Cell Physiol **298**(2): C263-273.

Husser, D., M. Stridh, L. Sornmo, D. M. Roden, D. Darbar and A. Bollmann (2009). "A genotype-dependent intermediate ECG phenotype in patients with persistent lone atrial fibrillation genotype ECG-phenotype correlation in atrial fibrillation." Circ Arrhythm Electrophysiol **2**(1): 24-28.

Isbrandt, D., P. Friederich, A. Solth, W. Haverkamp, A. Ebneth, M. Borggrefe, H. Funke, K. Sauter, G. Breithardt, O. Pongs and E. Schulze-Bahr (2002). "Identification and functional characterization of a novel KCNE2 (MiRP1) mutation that alters HERG channel kinetics." J Mol Med (Berl) **80**(8): 524-532.

Kaab, S., D. C. Crawford, M. F. Sinner, E. R. Behr, P. J. Kannankeril, A. A. Wilde, C. R. Bezzina, E. Schulze-Bahr, P. Guicheney, N. H. Bishopric, R. J. Myerburg, J. J. Schott, A. Pfeufer, B. M. Beckmann, E. Martens, T. Zhang, B. Stallmeyer, S. Zumhagen, I. Denjoy, A. Bardai, I. C. Van Gelder, Y. Jamshidi, C. Dalageorgou, V. Marshall, S. Jeffery, S. Shakir, A. J. Camm, G. Steinbeck, S. Perz, P. Lichtner, T. Meitinger, A. Peters, H. E. Wichmann, C. Ingram, Y. Bradford, S. Carter, K. Norris, M. D. Ritchie, A. L. George, Jr. and D. M. Roden (2012). "A large candidate gene survey identifies the KCNE1 D85N polymorphism as a possible modulator of drug-induced torsades de pointes." Circ Cardiovasc Genet **5**(1): 91-99.

Kapplinger, J. D., D. J. Tester, B. A. Salisbury, J. L. Carr, C. Harris-Kerr, G. D. Pollevick, A. A. M. Wilde and M. J. Ackerman (2009). "Spectrum and prevalence of mutations from the first 2,500 consecutive unrelated patients referred for the FAMILION® long QT syndrome genetic test." Heart Rhythm **6**(9): 1297-1303.

Lai, L.-P., Y.-N. Su, F.-T. Chiang, J.-M. Juang, Y.-B. Liu, Y.-L. Ho, W.-J. Chen, S.-J. Yeh, C.-C. Wang, Y.-L. Ko, T.-J. Wu, K.-C. Ueng, M.-H. Lei, H.-M. Tsao, S.-A. Chen, T.-K. Lin, M.-H. Wu, H.-M. Lo, S. K. S. Huang and J.-L. Lin (2005). "Denaturing high-performance liquid chromatography screening of the long QT syndrome-related cardiac sodium and potassium channel genes and identification of novel mutations and single nucleotide polymorphisms." J Hum Genet **50**(9): 490-496.

Lin, L., H. Horigome, N. Nishigami, S. Ohno, M. Horie and R. Sumazaki (2012). "Drug-induced QT-interval prolongation and recurrent torsade de pointes in a child with heterotaxy syndrome and KCNE1 D85N polymorphism." J Electrocardiol **45**(6): 770-773.

Ma, L., C. Lin, S. Teng, Y. Chai, R. Bahring, V. Vardanyan, L. Li, O. Pongs and R. Hui (2003). "Characterization of a novel Long QT syndrome mutation G52R-KCNE1 in a Chinese family." Cardiovasc Res **59**(3): 612-619.

Millat, G., P. Chevalier, L. Restier-Miron, A. Da Costa, P. Bouvagnet, B. Kugener, L. Fayol, C. Gonzalez Armengod, B. Oddou, V. Chanavat, E. Froidefond, R. Perraudin, R. Rousson and C. Rodriguez-Lafrasse (2006). "Spectrum of pathogenic mutations and associated polymorphisms in a cohort of 44 unrelated patients with long QT syndrome." Clin Genet **70**(3): 214-227.

Nakajima, T., J. Wu, Y. Kaneko, T. Ashihara, S. Ohno, T. Irie, W. G. Ding, H. Matsuura, M. Kurabayashi and M. Horie (2012). "KCNE3 T4A as the genetic basis of Brugada-pattern electrocardiogram." Circ J **76**(12): 2763-2772.

Napolitano, C., S. G. Priori, P. J. Schwartz, R. Bloise, E. Ronchetti, J. Nastoli, G. Bottelli, M. Cerrone and S. Leonardi (2005). "Genetic testing in the long QT syndrome: development and validation of an efficient approach to genotyping in clinical practice." JAMA **294**(23): 2975-2980.

Nielsen, N. H., B. G. Winkel, J. K. Kanters, N. Schmitt, J. Hofman-Bang, H. S. Jensen, B. H. Bentzen, B. Sigurd, L. A. Larsen, P. S. Andersen, S. Haunso, K. Kjeldsen, M. Grunnet, M. Christiansen and S. P. Olesen (2007). "Mutations in the Kv1.5 channel gene KCNA5 in cardiac arrest patients." Biochem Biophys Res Commun **354**(3): 776-782.

Nishio, Y., T. Makiyama, H. Itoh, T. Sakaguchi, S. Ohno, Y. Z. Gong, S. Yamamoto, T. Ozawa, W. G. Ding, F. Toyoda, M. Kawamura, M. Akao, H. Matsuura, T. Kimura, T. Kita and M. Horie (2009). "D85N, a KCNE1 polymorphism, is a disease-causing gene variant in long QT syndrome." J Am Coll Cardiol **54**(9): 812-819.

Ohno, S., F. Toyoda, D. P. Zankov, H. Yoshida, T. Makiyama, K. Tsuji, T. Honda, K. Obayashi, H. Ueyama and W. Shimizu (2009). "Novel KCNE3 mutation reduces repolarizing potassium current and associated with long QT syndrome." Human mutation **30**(4): 557-563.

Ohno, S., D. P. Zankov, W. G. Ding, H. Itoh, T. Makiyama, T. Doi, S. Shizuta, T. Hattori, A. Miyamoto, N. Naiki, J. C. Hancox, H. Matsuura and M. Horie (2011). "KCNE5 (KCNE1L) variants are novel modulators of Brugada syndrome and idiopathic ventricular fibrillation." Circ Arrhythm Electrophysiol **4**(3): 352-361.

Ohno, S., D. P. Zankov, H. Yoshida, K. Tsuji, T. Makiyama, H. Itoh, M. Akao, J. C. Hancox, T. Kita and M. Horie (2007). "N- and C-terminal KCNE1 mutations cause distinct phenotypes of long QT syndrome." Heart Rhythm **4**(3): 332-340.

Olesen, M. S., B. H. Bentzen, J. B. Nielsen, A. B. Steffensen, J. P. David, J. Jabbari, H. K. Jensen, S. Haunso, J. H. Svendsen and N. Schmitt (2012). "Mutations in the potassium channel subunit KCNE1 are associated with early-onset familial atrial fibrillation." BMC Med Genet **13**: 24.

Paulussen, A. D., R. A. Gilissen, M. Armstrong, P. A. Doevendans, P. Verhasselt, H. J. Smeets, E. Schulze-Bahr, W. Haverkamp, G. Breithardt, N. Cohen and J. Aerssens (2004). "Genetic variations of KCNQ1, KCNH2, SCN5A, KCNE1, and KCNE2 in drug-induced long QT syndrome patients." J Mol Med (Berl) **82**(3): 182-188.

Porthan, K., A. Marjamaa, M. Viitasalo, H. Vaananen, A. Jula, L. Toivonen, M. S. Nieminen, C. Newton-Cheh, V. Salomaa, K. Kontula and L. Oikarinen (2010). "Relationship of common candidate gene variants to electrocardiographic T-wave peak to T-wave end interval and T-wave morphology parameters." Heart Rhythm **7**(7): 898-903.

Prystupa, A., G. Dzida, W. Myslinski, G. Malaj and T. Lorenc (2006). "MinK gene polymorphism in the pathogenesis of lone atrial fibrillation." Kardiol Pol **64**(11): 1205-1211; discussion 1212-1203.

Ravn, L. S., Y. Aizawa, G. D. Pollevick, J. Hofman-Bang, J. M. Cordeiro, U. Dixen, G. Jensen, Y. Wu, E. Burashnikov, S. Haunso, A. Guerchicoff, D. Hu, J. H. Svendsen, M. Christiansen and C. Antzelevitch (2008). "Gain of function in IKs secondary to a mutation in KCNE5 associated with atrial fibrillation." Heart Rhythm **5**(3): 427-435.

Ravn, L. S., J. Hofman-Bang, U. Dixen, S. O. Larsen, G. Jensen, S. Haunso, J. H. Svendsen and M. Christiansen (2005). "Relation of 97T polymorphism in KCNE5 to risk of atrial fibrillation." Am J Cardiol **96**(3): 405-407.

Schulze-Bahr, E., M. Schwarz, S. Hauenschild, H. Wedekind, H. Funke, W. Haverkamp, G. Breithardt, O. Pongs and D. Isbrandt (2001). "A novel long-QT 5 gene mutation in the C-terminus (V109I) is associated with a mild phenotype." J Mol Med (Berl) **79**(9): 504-509.

Schulze-Bahr, E., Q. Wang, H. Wedekind, W. Haverkamp, Q. Chen, Y. Sun, C. Rubie, M. Hordt, J. A. Towbin, M. Borggrefe, G. Assmann, X. Qu, J. C. Somberg, G. Breithardt, C. Oberti and H. Funke (1997). "KCNE1 mutations cause jervell and Lange-Nielsen syndrome." Nat Genet **17**(3): 267-268.

Sesti, F., G. W. Abbott, J. Wei, K. T. Murray, S. Saksena, P. J. Schwartz, S. G. Priori, D. M. Roden, A. L. George, Jr. and S. A. Goldstein (2000). "A common polymorphism associated with antibiotic-induced cardiac arrhythmia." Proc Natl Acad Sci U S A **97**(19): 10613-10618.

Sesti, F. and S. A. N. Goldstein (1998). "Single-Channel Characteristics of Wild-Type IKs Channels and Channels formed with Two MinK Mutants that Cause Long QT Syndrome." The Journal of General Physiology **112**(6): 651-663.

Shim, S. H., M. Ito, T. Maher and A. Milunsky (2005). "Gene sequencing in neonates and infants with the long QT syndrome." Genet Test **9**(4): 281-284.

Splawski, I., J. Shen, K. W. Timothy, M. H. Lehmann, S. Priori, J. L. Robinson, A. J. Moss, P. J. Schwartz, J. A. Towbin, G. M. Vincent and M. T. Keating (2000). "Spectrum of Mutations in Long-QT Syndrome Genes: KVLQT1, HERG, SCN5A, KCNE1, and KCNE2." Circulation **102**(10): 1178-1185.

Splawski, I., M. Tristani-Firouzi, M. H. Lehmann, M. C. Sanguinetti and M. T. Keating (1997). "Mutations in the hminK gene cause long QT syndrome and suppress IKs function." Nat Genet **17**(3): 338-340.

Westenskow, P., I. Splawski, K. W. Timothy, M. T. Keating and M. C. Sanguinetti (2004). "Compound mutations: a common cause of severe long-QT syndrome." Circulation **109**(15): 1834-1841.

Wu, D. M., L. P. Lai, M. Zhang, H. L. Wang, M. Jiang, X. S. Liu and G. N. Tseng (2006). "Characterization of an LQT5-related mutation in KCNE1, Y81C: implications for a role of KCNE1 cytoplasmic domain in IKs channel function." Heart Rhythm **3**(9): 1031-1040.

Xu, L. X., W. Y. Yang, H. Q. Zhang, Z. H. Tao and C. C. Duan (2008). "[Study on the correlation between CETP TaqIB, KCNE1 S38G and eNOS T-786C gene polymorphisms for predisposition and non-valvular atrial fibrillation]." Zhonghua Liu Xing Bing Xue Za Zhi **29**(5): 486-492.

Yang, Y., M. Xia, Q. Jin, S. Bendahhou, J. Shi, Y. Chen, B. Liang, J. Lin, Y. Liu, B. Liu, Q. Zhou, D. Zhang, R. Wang, N. Ma, X. Su, K. Niu, Y. Pei, W. Xu, Z. Chen, H. Wan, J. Cui, J. Barhanin and Y. Chen (2004). "Identification of a KCNE2 gain-of-function mutation in patients with familial atrial fibrillation." Am J Hum Genet **75**(5): 899-905.

Zeng, Z., C. Tan, S. Teng, J. Chen, S. Su, X. Zhou, F. Wang, S. Zhang, D. Gu, J. C. Makielski and J. Pu (2007). "The single nucleotide polymorphisms of I(Ks) potassium channel genes and their association with atrial fibrillation in a Chinese population." Cardiology **108**(2): 97-103.

Zeng, Z. Y., J. L. Pu, C. Tan, S. Y. Teng, J. H. Chen, S. Y. Su, X. Y. Zhou, S. Zhang, Y. S. Li, F. Z. Wang and D. F. Gu (2005). "[The association of single nucleotide polymorphism of slow delayed rectifier K+ channel genes with atrial fibrillation in Han nationality Chinese]." Zhonghua Xin Xue Guan Bing Za Zhi **33**(11): 987-991.

Zhang, D.-F., B. Liang, J. Lin, B. Liu, Q.-S. Zhou and Y.-Q. Yang (2005). "KCNE3 R53H substitution in familial atrial fibrillation." CHINESE MEDICAL JOURNAL-BEIJING-ENGLISH EDITION- **118**(20): 1735.
